# Performance of the Monoclonal Antibody B72.3 in Diagnosis of Malignant Carcinomatous Serous Effusions—A Systematic Review and Meta‐Analysis of Diagnostic Performance

**DOI:** 10.1111/cyt.13493

**Published:** 2025-04-10

**Authors:** Alex H. Lin, Matthew Hsu, Joanna K. M. Ng, Sahar J. Farahani, Joshua J. X. Li, Jana Nano, Hamidreza Raeisi‐Dehkordi, Wilson Tang, Philippe Vielh, Taulant Muka

**Affiliations:** ^1^ Department of Pathology, School of Clinical Medicine The University of Hong Kong Pokfulam Hong Kong; ^2^ Department of Pathology and Laboratory Medicine Memorial Sloan Kettering Cancer Center New York USA; ^3^ Department of Radiation Oncology, TUM School of Medicine and Health, Klinikum Rechts der Isar Technical University of Munich Munich Germany; ^4^ Department of Global Public Health and Bioethics, Julius Center for Health Sciences and Primary Care University Medical Center (UMC) Utrecht, Utrecht University Utrecht the Netherlands; ^5^ The University of Hong Kong Libraries The University of Hong Kong Pokfulam Hong Kong; ^6^ Department of Pathology Medipath and American Hospital of Paris Paris France; ^7^ Epistudia Bern Switzerland

**Keywords:** B72.3, diagnosis, immunocytochemistry, meta‐analysis, metastatic carcinoma, serous effusion

## Abstract

**Objectives:**

Immunocytochemistry is often required in the cytologic assessment of malignant serous effusion, particularly for differentiating metastatic carcinoma from mesothelioma. To summarise the diagnostic performance of the monoclonal antibody B72.3, a systematic review and meta‐analysis was conducted.

**Methods:**

Five databases were searched for relevant studies and reviewed for data extraction and risk of bias assessment. Pooled sensitivity, specificity, positive likelihood ratio (PLR), negative likelihood ratio (NLR), diagnostic odds ratio (DOR) and area under curve of summary receiver operating characteristics (AUC‐SROC) were calculated for the diagnostic performance of B72.3. Heterogeneity and publication bias were assessed by the *I* ^*2*^ index and Deeks' funnel plot.

**Results:**

In total, 19 studies (1159 cases) were included. Overall pooled sensitivity and specificity were 0.76 (0.72–0.79) and 0.90 (0.74–1.00), respectively. The NLR, PLR and DOR were 0.27 (0.21–0.34), 7.66 (< 0.001–20.46) and 28.26 (0–75.96), respectively. The AUC‐SROC was 0.98, indicating a good overall diagnostic accuracy for B72.3. Subgroup analysis for adenocarcinoma (0.75, 0.71–0.79), mesothelioma (0.92, 0.85–0.98) and benign/reactive mesothelial cells (0.96, 0.93–1.00) showed similar sensitivity and specificity, while the sensitivity for adenocarcinomas of the gastrointestinal/hepatobiliary tract (0.56, 0.41–0.71) and breast (0.55, 0.38–0.71) was significantly lower. High heterogeneity was observed in the majority of our analyses, while no evidence of publication bias was identified.

**Conclusions:**

B72.3 has an acceptable performance with low sensitivity. With a good specificity, B72.3 may find use in an immunocytochemical panel for excluding benign mesothelial processes.

## Graphical abstract

B72.3 is a carcinoma marker with an acceptable performance but relatively low sensitivity. With its specificity, B72.3 may find use in an immunocytochemical panel for excluding benign mesothelial processes.

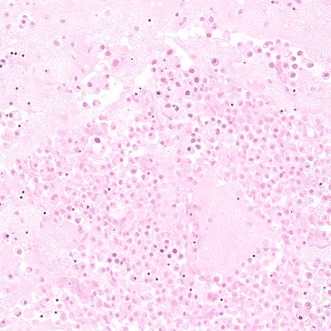

## 1 Introduction

Serous effusions, the abnormal accumulation of fluid within the serous cavities of the body, are frequently caused by malignant neoplasms involving bodily cavities, of which metastatic carcinomas are the most common and followed by mesothelioma, a malignancy originating from the mesothelial cells lining the serous cavities [1]. The diagnosis of carcinomatous serous effusion is often reliant on immunocytochemical markers and is of clinical significance due to the association with poor prognosis and indication of cancer therapy [2]. B72.3 is a monoclonal antibody against tumour‐associated glycoprotein 72 (TAG‐72), which is expressed on the surface of carcinoma cells [3]. There have been investigations on the prognostic and theragnostic role of B72.3, with evidence suggesting correlation between TAG‐72 expression and prognosis in gastrointestinal carcinomas [4, 5, 6] and the development of monoclonal antibodies targeting TAG‐72 [7]. Although these applications of B72.3/TAG‐72 have fallen out of favour, the B72.3 remains an accepted diagnostic marker. B72.3 has been extensively tested on cytological preparations for the purpose of distinguishing carcinoma cells from mesothelial cells (both mesothelioma and benign or reactive mesothelial cells) [8]. B72.3 is often used as a part of an immunocytochemical panel, including antibodies that are also immunoreactive in carcinoma cells [9, 10] and in the opposite antibodies that are expressed in mesothelial cells [11].

Although numerous studies have investigated the diagnostic performance of B72.3, the reported sensitivity, specificity and predictive values vary considerably, likely due to differences in study design, sample size and methodological approaches. Additionally, its diagnostic accuracy may vary based on the primary organ of adenocarcinoma or in the setting of mesothelioma. A comprehensive synthesis of this evidence, including subgroup analyses, is therefore essential to understand the diagnostic performance and role of B72.3 in clinical practice, with the introduction of newer carcinoma markers that serve as potential alternatives [10]. This meta‐analysis aims to comprehensively review the diagnostic accuracy and related metrics of B72.3 for the diagnosis of malignant carcinomatous serous effusions, with subgroup analysis on its performance on the diagnosis of adenocarcinomas, mesotheliomas, benign mesothelial cells and further subtypes of adenocarcinomas by organ primary.

## 2 Methods

### 2.1 Study Design

The study was designed and conducted following recent guidelines on how to perform a systematic review and meta‐analysis [12, 13], while the Preferred Reporting Items for Systematic Reviews and Meta‐Analyses (PRISMA) flowchart was used for reporting and the PRISMA for diagnostic test accuracy (PRISMA‐DTA) checklist was followed (Supporting Information S1).

### 2.2 Literature Search

Literature search of articles up to 30 June 2024 was performed, using the bibliographic databases Embase, Medline, PubMed, Scopus and Web of Science. Key medical subject headings and search terms related to both the biomarker and outcome were used such as ‘B72.3’, ‘cytology’, ‘effusion’, ‘fluid’, ‘sensitivity’, ‘specificity’, ‘accuracy’ and ‘diagnosis’. The full search strategy is provided in detail in the Supporting Information S2. Reference lists of the final included studies in the systematic review were screened for eligible studies. Endnote (version 20.1) was used for reference management.

### 2.3 Study Selection

Titles and abstracts of identified articles were screened by two independent reviewers (JL and JN) against predefined inclusion and exclusion criteria and settled discrepancies with discussion or with the opinion of a third reviewer. Studies were deemed eligible if (i) they were designed as randomised clinical trials, retrospective or prospective cohort, case–control or cross‐sectional studies or case series; (ii) had sample size > 15; (iii) they assessed the diagnostic accuracy of immunocytochemical marker B72.3 in the diagnosis of carcinoma in effusion fluid cytology; (iv) they reported the frequency of cases and provided histology and/or clinical follow‐up data which were regarded as the (combined) reference outcome. Articles were not eligible for inclusion if (i) they were non‐English, review articles, conference abstracts, letters to the editor, editorials or case reports; (ii) they combined histology or aspiration cytology specimens and that the results of the cytology specimens could not be extracted; (iii) combined non‐carcinomatous malignant effusions such as lymphoma, melanoma and sarcoma, and that the results of the carcinomatous malignant effusions could not be extracted.

### 2.4 Data Extraction and Quality Assessment

The data extraction sheet included study methodology, population characteristics, setting, technical and diagnostic descriptions of cytologic preparation and immunocytochemistry procedures, reference standard and test results. For randomised clinical trials and prospective cohort studies, only the baseline or incidence assessment was used. Data extraction was performed by two independent reviewers (AL and JL), and all discrepancies were settled by discussion until a consensus was reached. The QUADAS‐2 (Quality Assessment of Diagnostic Accuracy Studies‐2) [14] was used for risk of bias and applicability assessment of the included studies.

### 2.5 Statistical Analysis

Extracted test result data (overall and subgroup case counts for true positive, true negative, false positive and false negative) were used to construct study specific two‐by‐two tables for calculation of diagnostic performance metrics including sensitivity, specificity, positive likelihood ratio (PLR), negative likelihood ratio (NLR) diagnostic odds ratio (DOR) and area under curve of summary receiver operating characteristics (AUC‐SROC). Results for each outcome were pooled using the DerSimonian and Laird random‐effects model. Diagnostic performance metrics for each subgroup of metastatic carcinomas classified by site of primary were also calculated. Statistical heterogeneity was assessed using Higgins' *I* ^*2*^ statistics, categorising *I* ^*2*^ values as follows: less than 25% indicated low heterogeneity, between 25% and 50% indicated moderate heterogeneity and greater than 50% indicated high heterogeneity. Publication bias was assessed by examination of the Deeks' funnel plot and Deeks' test statistic. Prespecified random‐effects meta‐regression and subgroup analyses were performed to investigate potential sources of heterogeneity (including the year of publication, sample size, sample composition of adenocarcinoma, quality of study and type of cytologic preparation). Analyses were performed using SPSS (Statistical Product and Service Solutions, version 20) and Python with the following packages—matplotlib, numpy, scipy, sklearn, statsmodels, pandas and pymare [15, 16, 17, 18, 19, 20, 21]. A p‐value of < 0.05 (two‐tailed) was considered statistically significant.

## 3 Results

### 3.1 Study Inclusion

**FIGURE 1:**
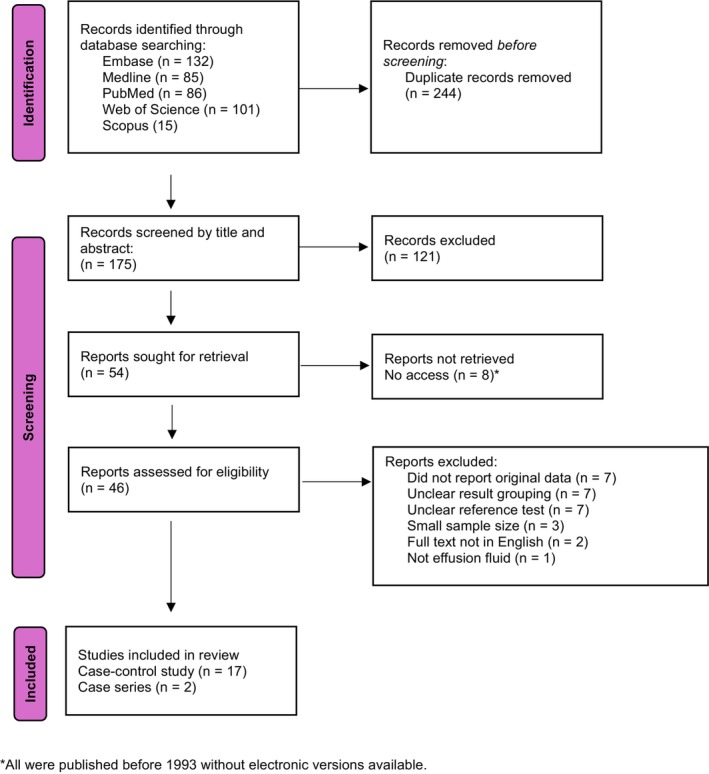
PRISMA flowchart. *All were published before 1993 without electronic versions available.

Following screening of 175 abstracts, 19 studies were included in the final analysis [8, 9, 22, 23, 24, 25, 26, 27, 28, 29, 30, 31, 32, 33, 34, 35, 36, 37, 38]. The selection process is summarised in the PRISMA flowchart (Figure 1). The sample size of the studies ranged from 19 to 154, totalling 1,159 cases. The majority of studies were conducted in the United States (*n* = 13), followed by the Netherlands (*n* = 2) and Norway (*n* = 2) (Tables 1 and 2). In terms of quality assessment, the most common risk of bias among the studies was that there was no blinding of the reference diagnosis to the assessors (*n* = 14), followed by a non‐consecutive case collection (*n* = 7) (Table 1). The detailed QUADAS‐2 assessment is presented in Table 1.

**TABLE 1:** Characteristics and risk of bias assessment of the studies included.

Study	Year	Institution	Case number (control)	Risk of bias			Applicability concerns			
				Patient selection	Index test	Reference standard	Flow and timing	Patient selection	Index test	Reference standard
Johnston et al. [8]	1985	USA	123 (41)	U	H	U	L	L	L	L
Martin et al. [22]	1986	USA	32 (4)	U	H	U	L	L	L	L
Szpak et al. [23]	1986	USA	40 (18)	H	L	U	L	L	L	L
Otis et al. [24]	1987	USA	33 (19)	H	L	U	L	H	L	L
Esteban et al. [25]	1990	USA	36 (18)	L	L	L	L	L	L	L
Nance et al. [26]	1991	USA	26 (3)	H	H	U	L	L	L	L
Shield et al. [27]	1994	Australia	153 (51)	L	H	U	L	L	L	L
Friedman et al. [28]	1996	USA	19 (15)	U	H	U	L	H	L	L
Delahaye et al. [29]	1997	Netherlands	154 (66)	L	H	U	L	L	L	L
Benevolo et al. [30]	1998	Italy	38 (0)	H	H	U	L	L	L	L
Davidson et al. [31]	1999	Norway	141 (83)	L	H	U	L	L	L	L
Lazcano et al. [32]	2000	USA	19 (0)	H	H	U	L	L	L	L
Ko et al. [33]	2001	USA	53 (24)	U	H	U	L	L	L	L
Mensch et al. [34]	2002	USA	19 (10)	H	H	U	L	L	L	L
Fetsch et al. [35]	2002	USA	29 (15)	U	H	U	L	L	L	L
Davidson et al. [36]	2002	Norway	88 (44)	U	H	L	L	L	L	L
Afify et al. [37]	2005	USA	64 (25)	L	L	U	L	L	L	L
Grefte et al. [38]	2008	Netherlands	34 (22)	H	L	U	L	L	L	L
Patel et al. [9]	2020	USA	58 (18)	L	H	L	L	L	L	L

Abbreviations: H, high risk; L, low risk; U, unclear risk.

**TABLE 2:** Technical specifications of B72.3 immunocytochemistry of the studies included.

Study	Year	Preparation	Source	Dilution	Intensity	Percentage	Pattern (membranous, nuclear, cytoplasm)
Johnston et al. [8]	1985	Cell block	NS	NS	NS	NS	Cytoplasmic/membraneous
Martin et al. [22]	1986	Liquid based	NS	NS	≥ 1+ (weak)	NS	Cytoplasmic/membraneous
Szpak et al. [23]	1986	Cell block	NS	NS	NS	≥ 10%	Cytoplasmic/membraneous
Otis et al. [24]	1987	Cell block	NS	NS	≥ 1+ (weak)	NS	NS
Esteban et al. [25]	1990	Cell block	Biomedical Technologies	1:1	NS	NS	NS
Nance et al. [26]	1991	Cell block	Biomedical Technologies	1:1	≥ 1+ (weak)	NS	NS
Shield et al. [27]	1994	Cell block	Biogenex	1:100	NS	NS	Cytoplasmic/membraneous
Friedman et al. [28]	1996	Liquid based	Signet Laboratories	NS	≥ 1+ (weak)	NS	NS
Delahaye et al. [29]	1997	Cell block	Triton Diagnostic	1:100	NS	NS	Cytoplasmic/membraneous
Benevolo et al. [30]	1998	Liquid based	Sorin Biomedica	NS	NS	≥ 10%	NS
Davidson et al. [31]	1999	Cell block	Biogenex	1:50	≥ 1+ (weak)	NS	Membranous
Lazcano et al. [32]	2000	Liquid based	Signet Laboratories	1:30	NS	NS	Predominant membranous ± cytoplasmic
Ko et al. [33]	2001	Cell block	Ventana	NS	NS	> 1%	Cytoplasmic
Mensch et al. [34]	2002	Liquid based	Biogenex	1:200	NS	NS	NS
Fetsch et al. [35]	2002	Cell block and liquid based^a^	Biogenex	1:100	NS	NS	Cytoplasmic/membraneous
Davidson et al. [36]	2002	Cell block	Biogenex	1:20	≥ 1+ (weak)	≥ 1%	NS
Afify et al. [37]	2005	Cell block	Biogenex	1:100	NS	≥ 10%	Cytoplasmic/membraneous
Grefte et al. [38]	2008	Cell block	Neomarkers/Labvision	1:1000	NS	≥ 50%	NS
Patel et al. [9]	2020	Cell block	Biogenex	NS	≥ 1+ (weak)	> 5%	Cytoplasmic/membraneous

Abbreviation: NS, not specified.

^a^ Staining was performed for all preparations in Fetsch et al.; the results from cell block staining were used for analysis.

### 3.2 Diagnostic Metrics

**FIGURE 2:**
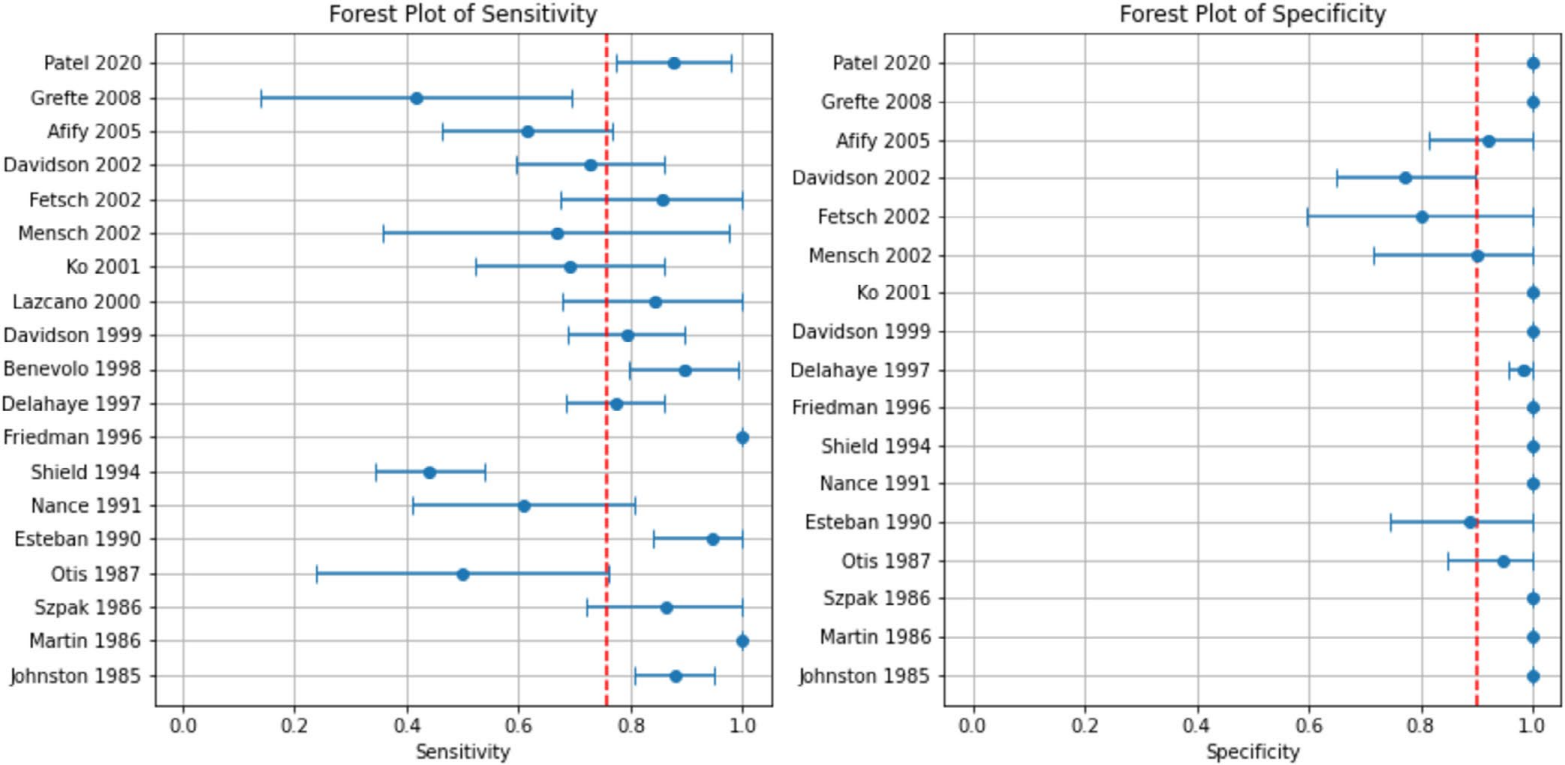
Forest plots of overall sensitivity and specificity.

**FIGURE 3:**
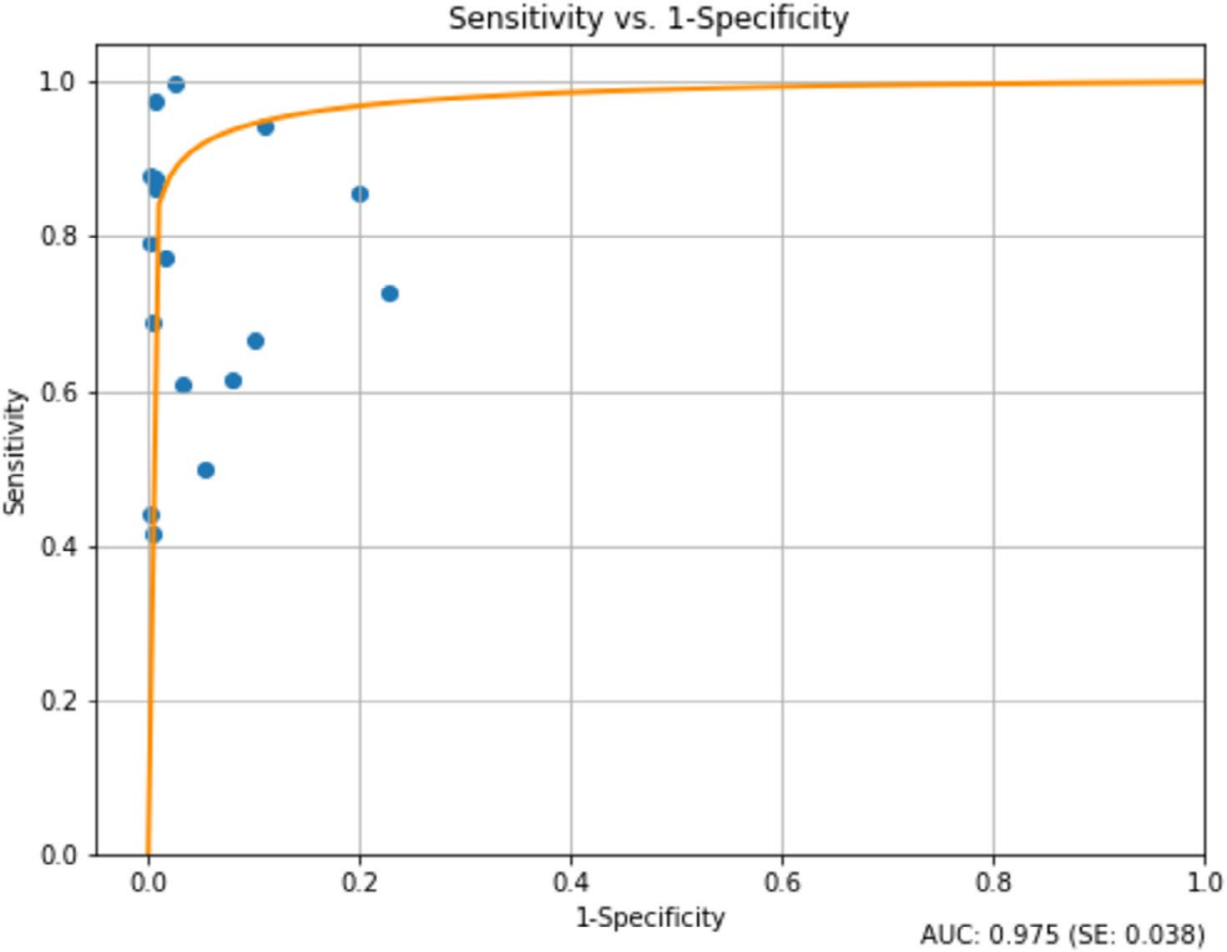
Area under curve of summary receiver operating characteristics (AUC‐SROC).

High levels of heterogeneity were observed for sensitivity, specificity, PLR, NLR and DOR (*I* ^*2*^ > 50%, *p* < 0.05); as such, the random effects model was used for calculation of values of pooled sensitivity, specificity, PLR, NLR and DOR. The overall pooled sensitivity and specificity were 0.76 (95% CI: 0.72–0.79) and 0.90 (95% CI: 0.74–1.00), respectively. The NLR, PLR and DOR were 0.27 (95% CI: 0.21–0.34), 7.66 (95% CI: < 0.001–20.46) and 28.26 (95% CI: 0–75.96), respectively. The forest plots of sensitivity and specificity are shown in Figure 2. The AUC‐SROC was 0.98, indicating a good overall diagnostic accuracy for B72.3 (Figure 3).

Subgroup analysis was performed for samples of adenocarcinoma, mesothelioma, and benign or reactive mesothelial cells. Similarly, subgroup analysis for sensitivity and specificity yielded high levels of heterogeneity (*I* ^*2*^ > 50%, *p* < 0.05) and the random effects model was used. The sensitivity for the adenocarcinoma subgroup was 0.75 (95% CI: 0.71–0.79), while the specificity for the mesothelioma and benign or reactive mesothelial cells was 0.92 (0.85–0.98) and 0.96 (95% CI: 0.93–1.00). There were 404 cases of metastatic adenocarcinoma with the site of primary and staining result retrievable, including from the most frequent to the least—female genital organs (*n* = 119), lung (*n* = 152), breast (*n* = 72), gastrointestinal/hepatobiliary tract (*n* = 58), kidney (*n* = 2), salivary gland (*n* = 1). Further subgroup analysis for sensitivity also showed high levels of heterogeneity (*I* ^*2*^ > 50%, *p* < 0.05) and the random effects model was used. The highest sensitivity was seen in metastatic adenocarcinomas of female genital primary (0.74, 95% CI: 0.63–0.84), followed by lung (0.70, 95% CI: 0.60–0.79), gastrointestinal/hepatobiliary tract (0.56, 95% CI: 0.41–0.71) and breast (0.55, 95% CI: 0.38–0.71) (Supporting Information S3).

Meta‐regression analysis showed only the year of publication to be a significant source of heterogeneity (*p* = 0.002) while other factors did not reach statistical significance (*p* > 0.05).

### 3.3 Publication Bias

**FIGURE 4:**
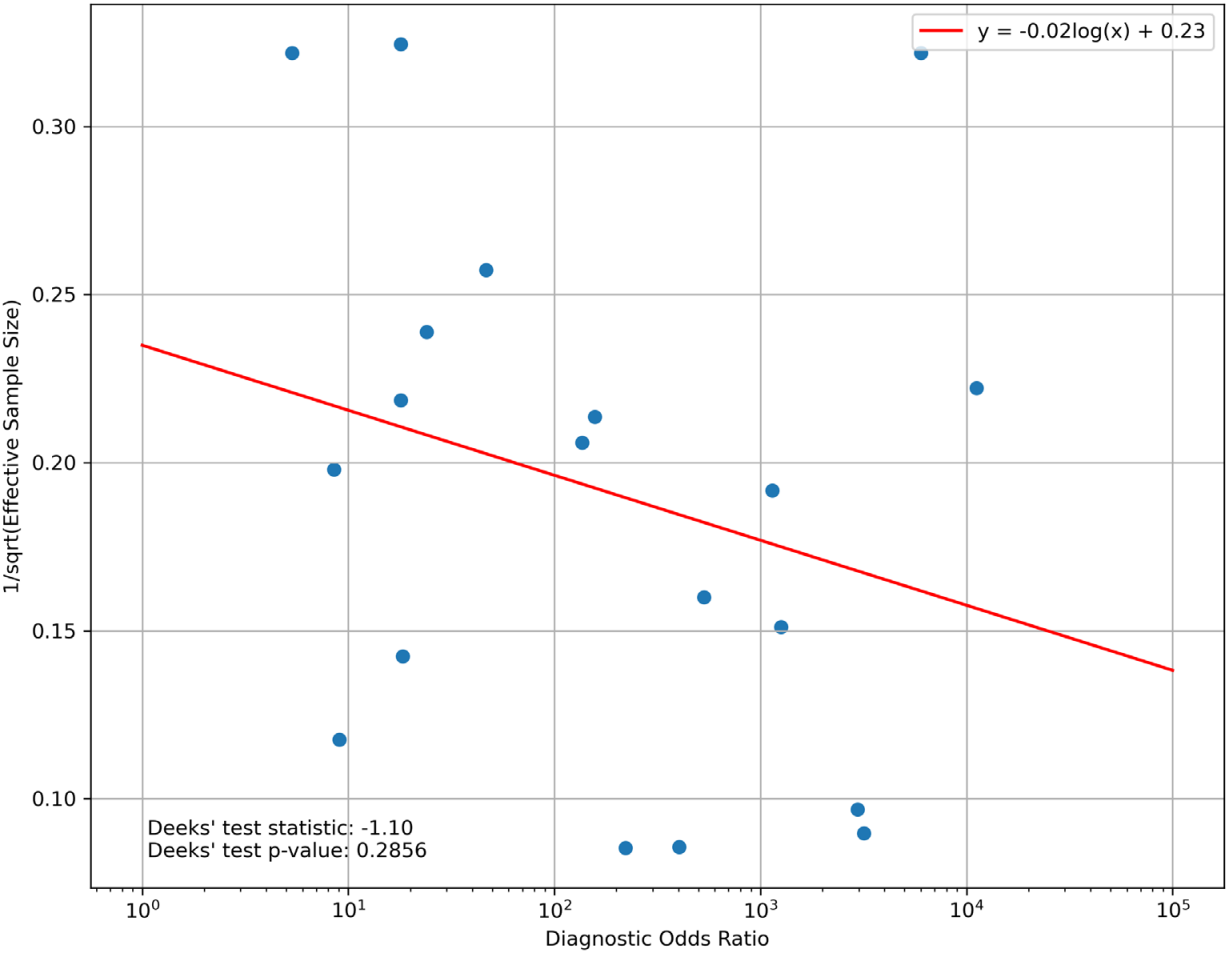
Deeks' funnel plot.

Examination of the Deeks' funnel plot did not show obvious asymmetry. The Deeks' test did not yield a significant p‐value (*p* = 0.29), indicating a low risk of publication bias (Figure 4).

## 4 Discussion

We demonstrated that B72.3 has an excellent overall diagnostic accuracy for malignant carcinomatous serous effusions, with a pooled sensitivity of 0.76, specificity of 0.90 and an AUC‐SROC of 0.98, while showing variable performance based on tumour subtype and primary site. These findings highlight the strong diagnostic utility of B72.3 in distinguishing malignant cells in serous effusions, particularly for adenocarcinomas, and underscore its potential role as a valuable tool in routine immunocytochemical panels to guide clinical decision making and improve diagnostic accuracy in challenging cases.

Serous effusion is a commonly encountered clinical presentation with a large range of underlying causes [39]. The most significant differentiation lies between benign serous effusion and malignant serous effusion. The majority of malignant serous effusions are due to metastatic carcinoma (i.e., malignant carcinomatous serous effusion), followed by mesothelioma [1]. Cytomorphologically, metastatic carcinoma and mesothelioma are readily distinguishable from other types of malignant cells, such as lymphoma, melanoma and sarcoma. Lymphomas are dispersed, with a high nuclear–cytoplasmic ratio and hyperchromatic nuclei; melanoma often contains pigment; and sarcomas are more often spindled without cohesive secondary structures [40, 41].

For the differential diagnosis between carcinoma and mesothelioma, it is difficult to achieve a definite cytologic diagnosis based on cytomorphology alone. Specific cytomorphological features, such as glandular formation for adenocarcinoma and giant atypical mesothelial cells for mesothelioma, may be used to support or favour a particular cytologic diagnosis [42]. These features only apply to adenocarcinomas on the well‐differentiated side of the spectrum and mesotheliomas that are well sampled. A definitive diagnosis for metastatic carcinoma or mesothelioma requires additional supporting evidence by ancillary testing, primarily immunocytochemistry and occasionally complemented by molecular tests [10, 43].

The AUC‐SROC of 0.98 indicated a good overall diagnostic performance for B72.3, slightly higher than data reported by meta‐analyses on BerEP4 [44] while lower than that of claudin‐4 [45]. The overall pooled sensitivity and specificity were 0.76 and 0.90 for B72.3, which were significantly lower than claudin‐4, at 0.98 and 1.00 in a meta‐analysis performed by Kleinaki et al. [45]. The sensitivity of BerEP4 and MOC31 was also reported to be > 0.8 [44, 46, 47], superior to B72.3. As for specificity, B72.3, BerEP4 and claudin‐4 exceeded 0.9, which may show an advantage over MOC31 with reported specificity as low as 0.6–0.7 [46, 48].

Carcinoma markers may not perform as well for certain types of carcinomas. For example, BerEP4 is known to be negative in squamous cell carcinomas, and thus less sensitive to carcinoma cells overall [49]. Subgroup analysis for adenocarcinoma with subtyping by organ primary, however, showed similar sensitivity for the adenocarcinoma subgroup (0.75), with similar to lower sensitivities for metastatic adenocarcinomas for individual organ primaries analysed (0.74–0.55). As for mesothelial cells, the sensitivity of B72.3 to benign mesothelial cells was higher than that for mesothelioma but did not reach statistical significance.

A high level of heterogeneity was observed between the studies included. Thus, meta‐regression was performed for identified year of publication as a contributing factor to heterogeneity. It should also be noted that a large proportion of studies included were conducted before the year 2000, with only one study published between 2020 and 2024 (June 30th, the end of the inclusion period). The standardisation, refinements and developments in immunocytochemical techniques, such as antigen retrieval, immunostaining and cell block (or equivalent) preparation [48, 50, 51] may have put B72.3 at a disadvantage compared to more recently popularised carcinoma markers. Such is evidenced by a relatively higher sensitivity (0.88) and specificity [1] from the most recently published article in the year 2020 by Patel et al. [9] of the current meta‐analysis. How dated protocols affected the performance is unclear, and the type of preparation (liquid‐based preparation vs. cell block) was not demonstrated as a factor in heterogeneity. Regardless, the principle and important steps in performing immunocytochemistry remain unchanged throughout the period.

## 5 Conclusion

As a carcinoma marker, despite an acceptable overall diagnostic performance, B72.3 suffers from a low sensitivity compared to other available carcinoma markers such as claudin‐4, MOC31 and BerEP4. The sensitivity of B72.3 is not improved in the subgroup for adenocarcinomas, including adenocarcinomas limited to a single/group of primary sites. The specificity of B72.3, however, exceeds 0.90 with a specificity of 0.96 to benign or reactive mesothelial cells. As a single marker, B72.3 is outperformed by most other carcinoma markers, but it may find a role in a diagnostic immunocytochemical panel, with an edge in excluding benign mesothelial processes.

## Author Contributions

**Alex H. Lin:** data curation, formal analysis, investigation, methodology, visualisation, writing – original draft. **Matthew Hsu:** formal analysis, methodology, validation. **Joanna K. M. Ng:** investigation, validation. **Sahar J. Farahani:** conceptualisation, methodology, validation. **Joshua J. X. Li:** conceptualisation, formal analysis, investigation, methodology, visualisation, writing – review and editing. **Jana Nano:** methodology, validation. **Hamidreza Raeisi‐Dehkordi:** methodology, validation. **Wilson Tang:** methodology, resources. **Philippe Vielh:** conceptualisation, methodology, supervision, validation, writing – review and editing. **Taulant Muka:** conceptualisation, methodology, supervision, validation, writing – review and editing.

## Conflicts of Interest

The authors declare no conflicts of interest.

## Supporting information


**Supporting Information S1.** PRISMA‐DTA checklist.

**Supporting Information S2.** Search algorithms.

**Supporting Information S3.** Forest plots of subgroup analyses.

## Data Availability

The data used to support the findings of this study are available from the corresponding author upon reasonable request.
