# Supplementary material for: Performance of the Monoclonal Antibody B72.3 in Diagnosis of Malignant Carcinomatous Serous Effusions—A Systematic Review and Meta‐Analysis of Diagnostic Performance
Source: Cytopathology. 2025 Apr 10;36(4):399–407. doi: 10.1111/cyt.13493 (PMC12150004; doi:10.1111/cyt.13493)
Supplement: Supplementary file 2 — Supporting Information S2. Search algorithms. [file CYT-36-399-s003.docx]

Supplementary material 2. Search algorithms

PubMed

("B72.3"[tiab]) AND (“cytology”[Mesh] OR “cytolog*”[tiab] OR “pleural effusion”[Mesh] OR “pericardial effusion”[Mesh] OR “peritoneal effusion”[Mesh] OR “effusion”[tiab] OR “fluid”[tiab]) AND ("sensitivity and specificity"[Mesh] OR "sensiti*"[tiab] OR "specifici*"[tiab] OR “accura*”[tiab] OR "diagnosis"[Mesh] OR "diagno*"[tiab])

WOS

("B72.3") AND (“cytology” OR “cytolog*” OR “pleural effusion” OR “pericardial effusion” OR “peritoneal effusion” OR “effusion” OR “fluid”) AND ("sensitivity and specificity" OR "sensiti*" OR "specifici*" OR “accura*” OR "diagnosis" OR "diagno*")

Medline

“B72.3”.mp. AND (Cytology/ or cytolog*.mp. or Pleural Effusion/ or Pericardial Effusion/ or Ascitic Fluid/ or effusion.mp. or fluid.mp.) AND ("Sensitivity and Specificity"/ or sensiti*.mp. or specifici*.mp. or accura*.mp. or Diagnosis/ or diagno*.mp.)

Scopus

(B72.3) AND (cytology OR cytolog* OR pleural effusion OR pericardial effusion OR peritoneal effusion OR effusion OR fluid) AND (sensitivity and specificity OR sensiti* OR specifici* OR accura* OR diagnosis OR diagno*)

Embase

“B72.3”.mp. AND (Cytology/ or cytolog*.mp. or Pleural Effusion/ or Pericardial Effusion/ or Ascitic Fluid/ or effusion.mp. or fluid.mp.) AND ("Sensitivity and Specificity"/ or sensiti*.mp. or specifici*.mp. or accura*.mp. or Diagnosis/ or diagno*.mp.)
