# Supplementary material for: Performance of the Monoclonal Antibody B72.3 in Diagnosis of Malignant Carcinomatous Serous Effusions—A Systematic Review and Meta‐Analysis of Diagnostic Performance
Source: Cytopathology. 2025 Apr 10;36(4):399–407. doi: 10.1111/cyt.13493 (PMC12150004; doi:10.1111/cyt.13493)

Supplementary material 3. Forest plots of subgroup analyses

a. Adenocarcinoma (sensitivity)


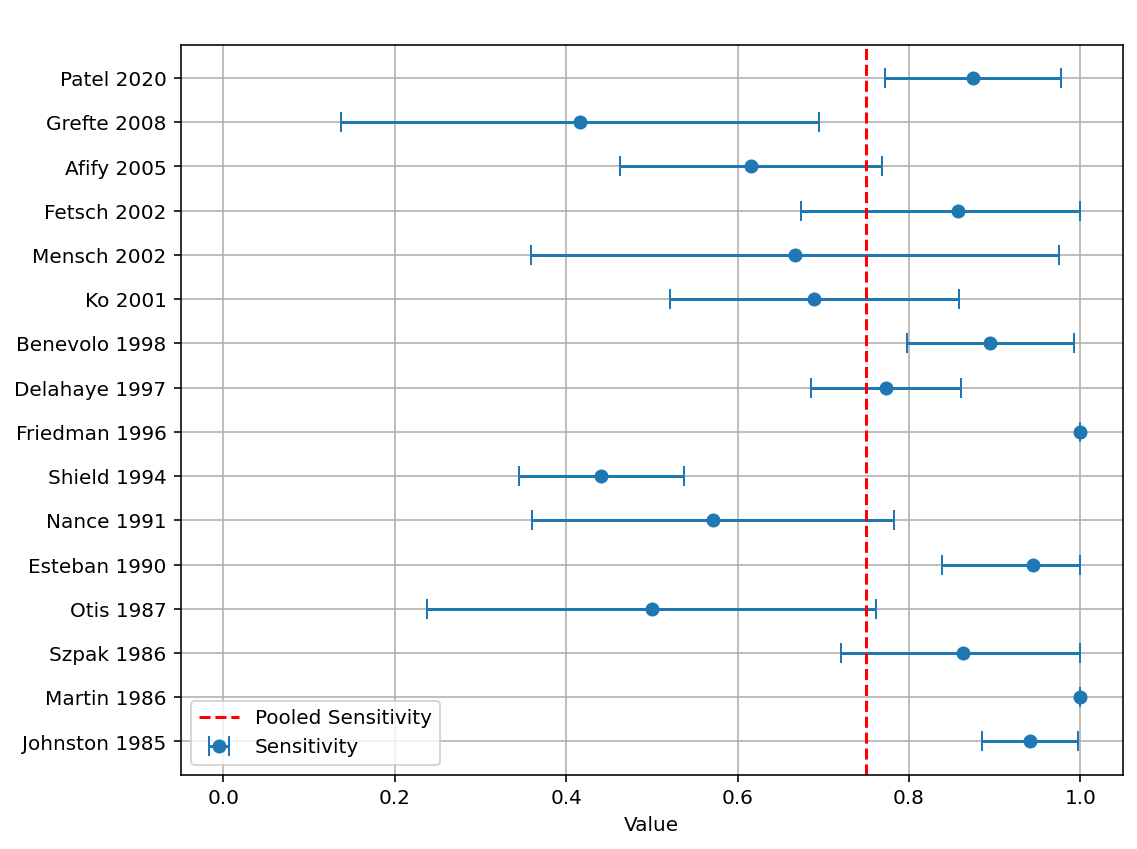


b. Mesothelioma (specificity)
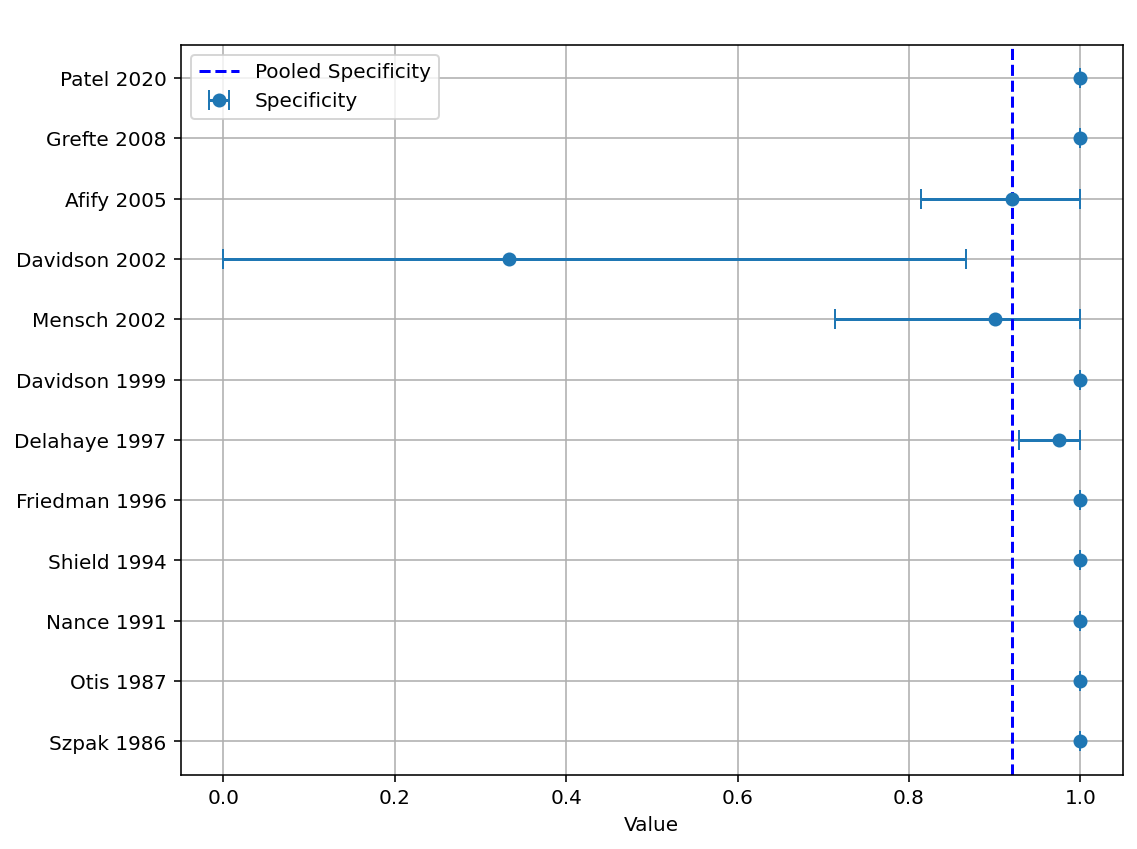


c. Benign/reactive mesothelial cells (specificity)


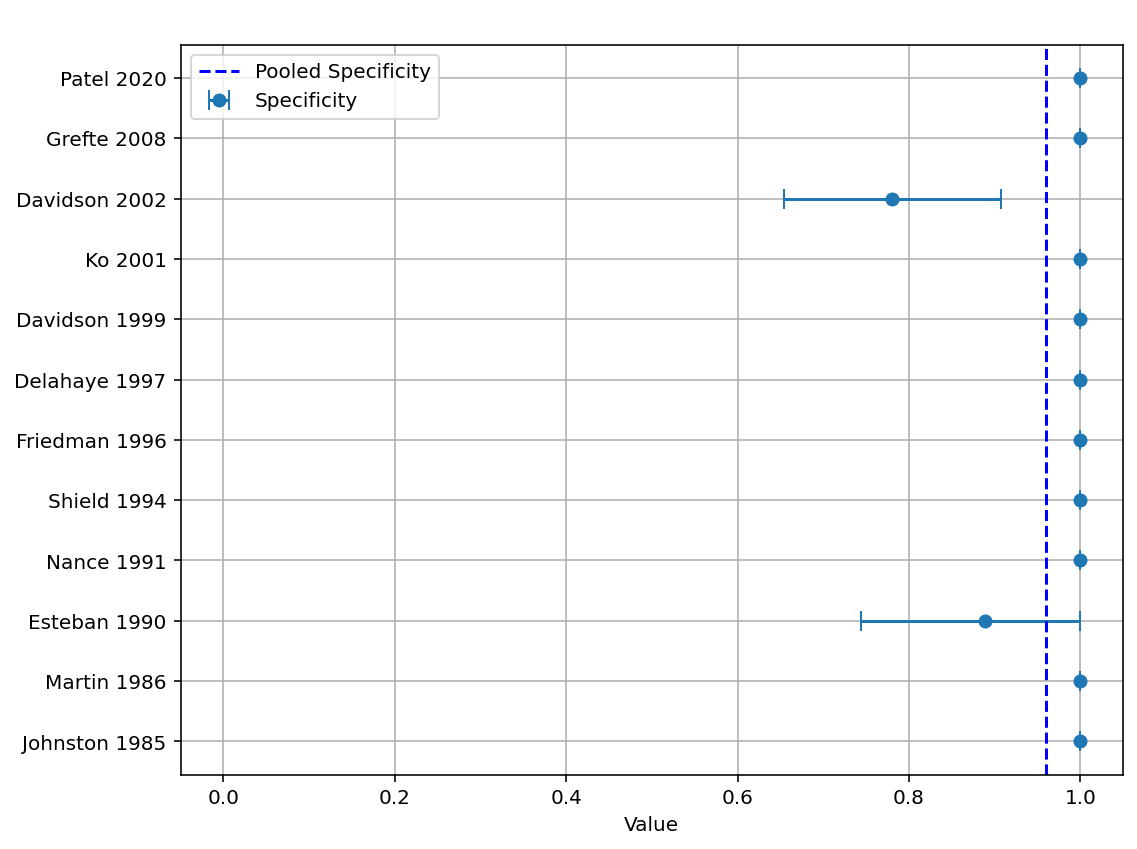


d. Female genital carcinomas (sensitivity)


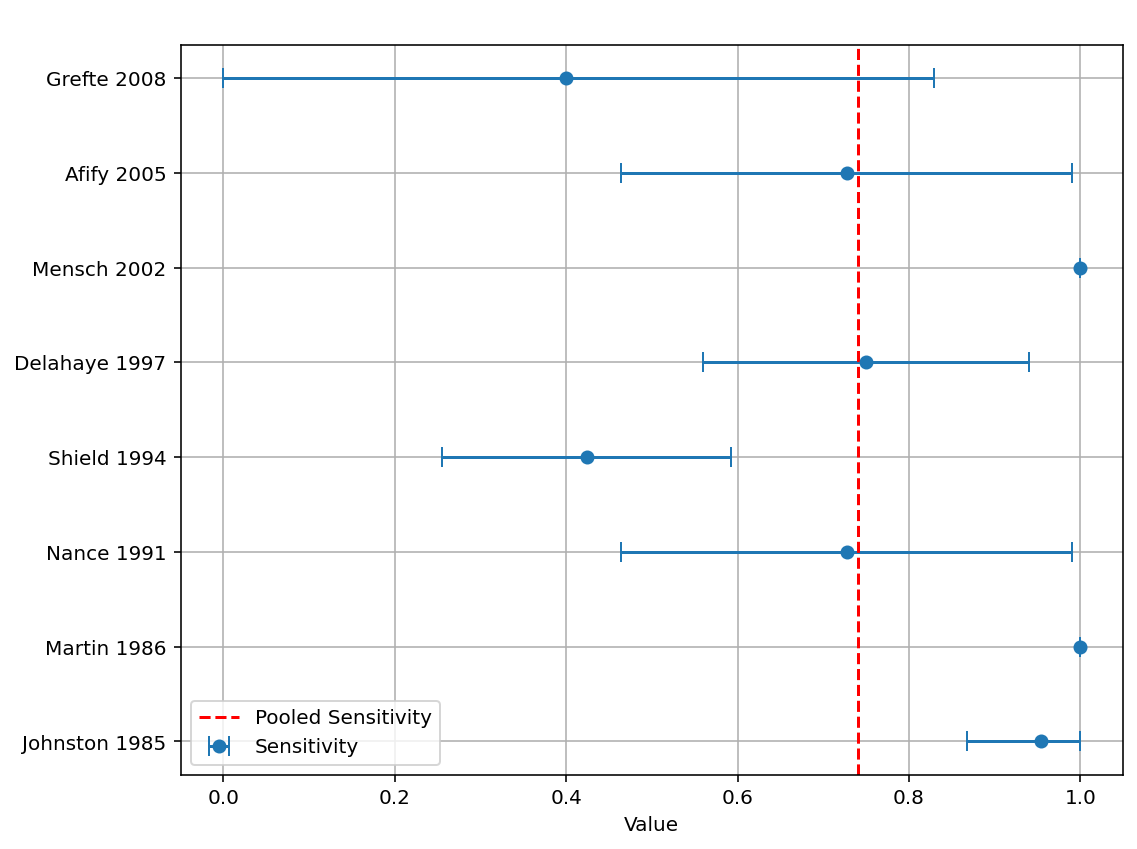


e. Lung carcinomas (sensitivity)


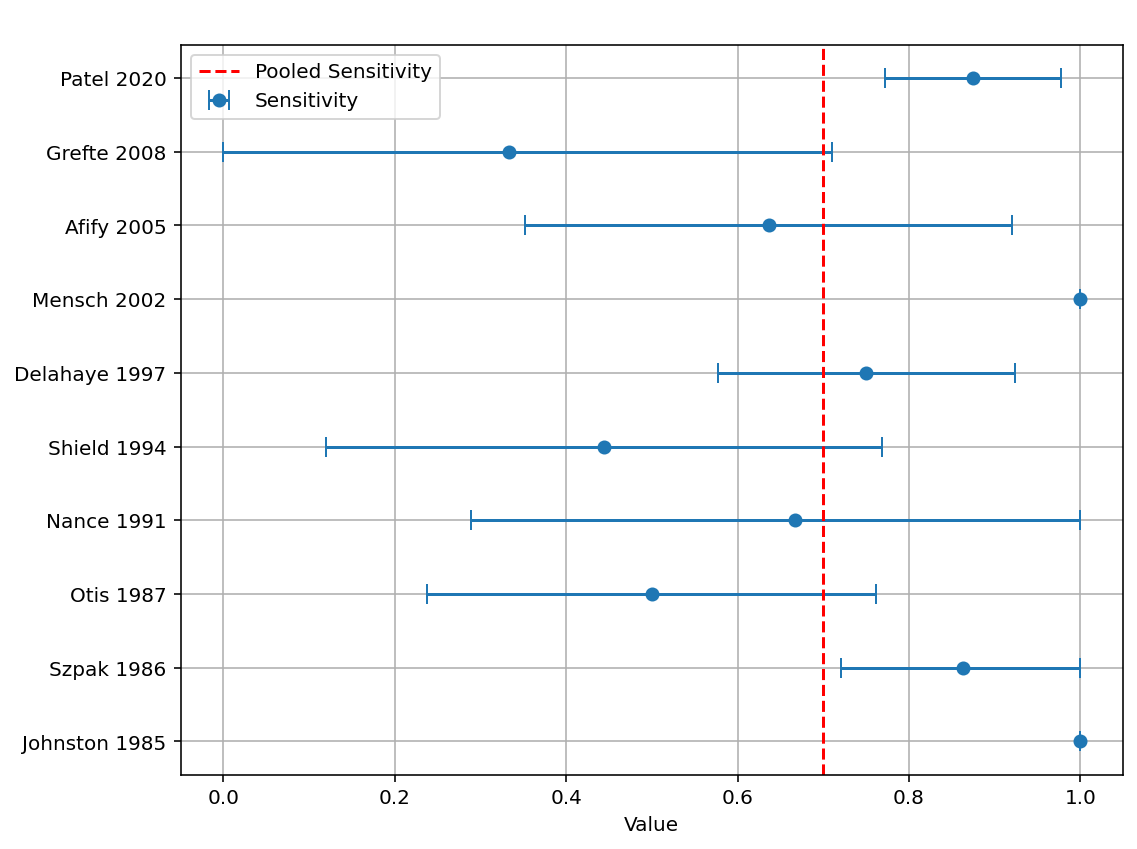


f. Breast carcinomas (sensitivity)
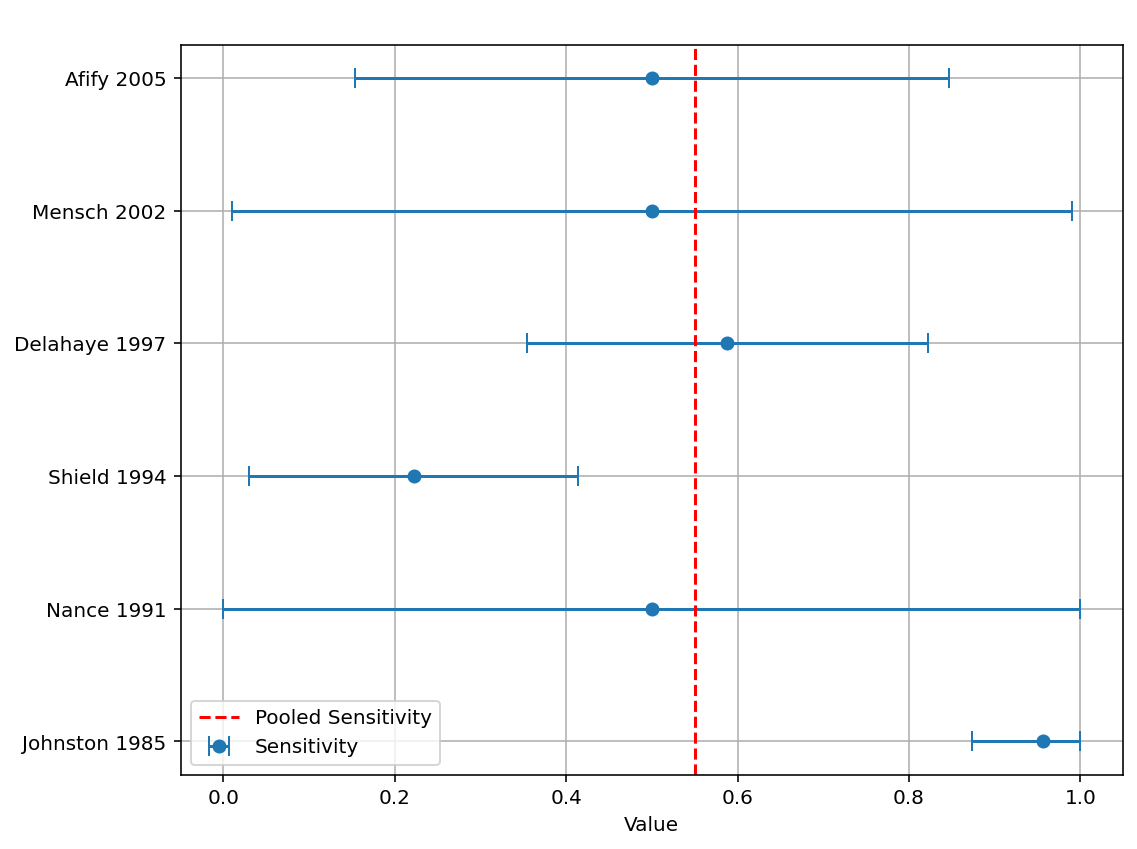


g. Gastrointestinal/hepatobiliary tract carcinomas (sensitivity)


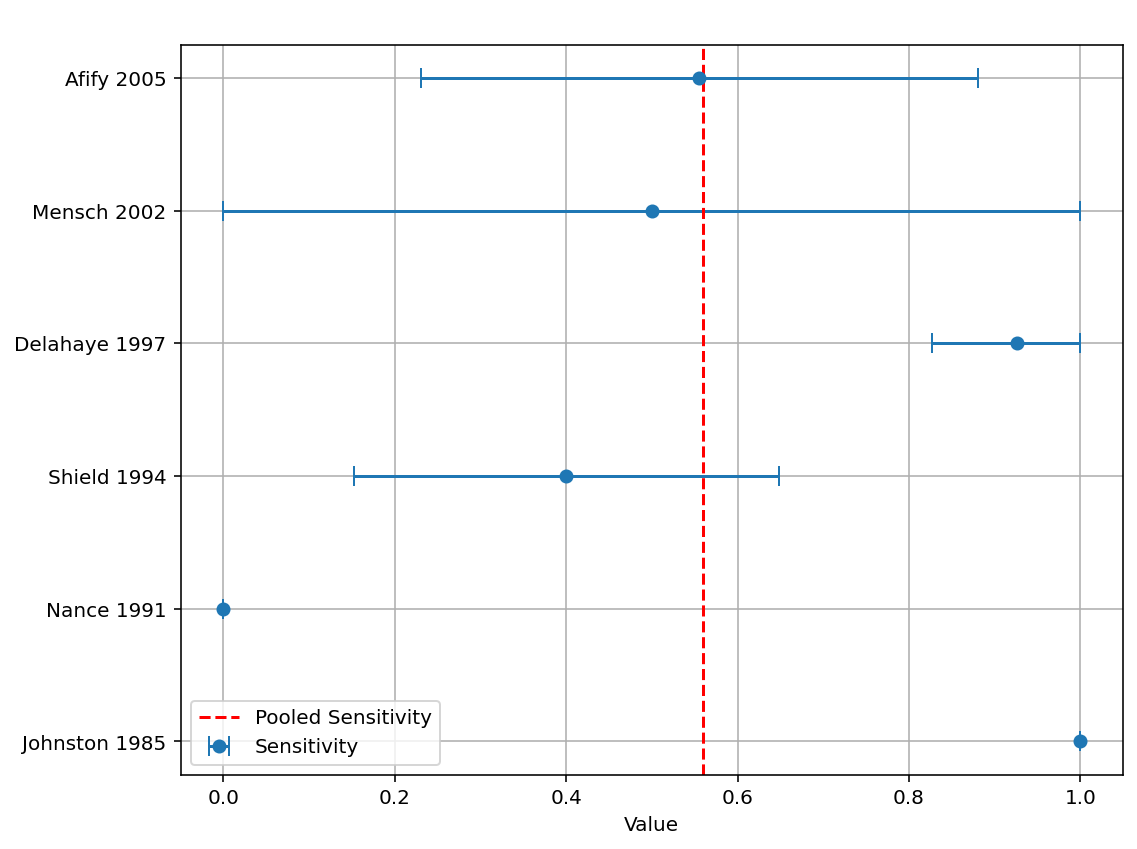

Supplement: Supplementary file 3 — Supporting Information S3. Forest plots of subgroup analyses. [file CYT-36-399-s002.docx]
